# Isolated zone I vertical fracture of first sacral vertebra: a case report

**DOI:** 10.1186/1757-1626-2-6254

**Published:** 2009-05-29

**Authors:** Serkan Bilgic, Volkan Kilincoglu, Yüksel Yurttas, Kenan Soylu, Ali Sehirlioglu

**Affiliations:** 1 Department of Orthopaedics and Traumatology, Gulhane Military Medical AcademyAnkaraTurkey; 2 Department of Radiology, Gulhane Military Medical AcademyAnkaraTurkey

## Abstract

Isolated sacral fractures which occur by shear forces on the pelvic ring are seen less commonly and they are commonly transversely oriented. A 29-year-old Turkish female patient, who sat in front seat in the car, was unrestrained, and another car hit them from right front side of their vehicle. Physical examination revealed considerable tenderness over the right superior gluteal region and excruciating pain during sacral and iliac compression. There was no clear fracture line in her plain radiographs. CT revealed incomplete, zone I fracture located on the superior and anterior part of the first sacral vertebra. Type 1 lateral compression pelvic fractures are relatively common and they include impacted sacral and ipsilateral rami fractures. Only a few cases, related with the isolated sacral fracture, have been reported in the literature. To our knowledge, no isolated vertical zone I fracture of the first sacral vertebra which occurred with the lateral compression injury has been described previously. Fracture of the sacrum should be suspected in the presence of sacral pain and tenderness.

## Introduction

Isolated sacral fractures which occur by shear forces on the pelvic ring are seen less commonly and they are commonly transversely oriented [1].

In our report, we described an Isolated zone I vertical fracture of first sacral vertebra which occurred with the lateral compression injury mechanism.

## Case presentation

**Figure 1.**
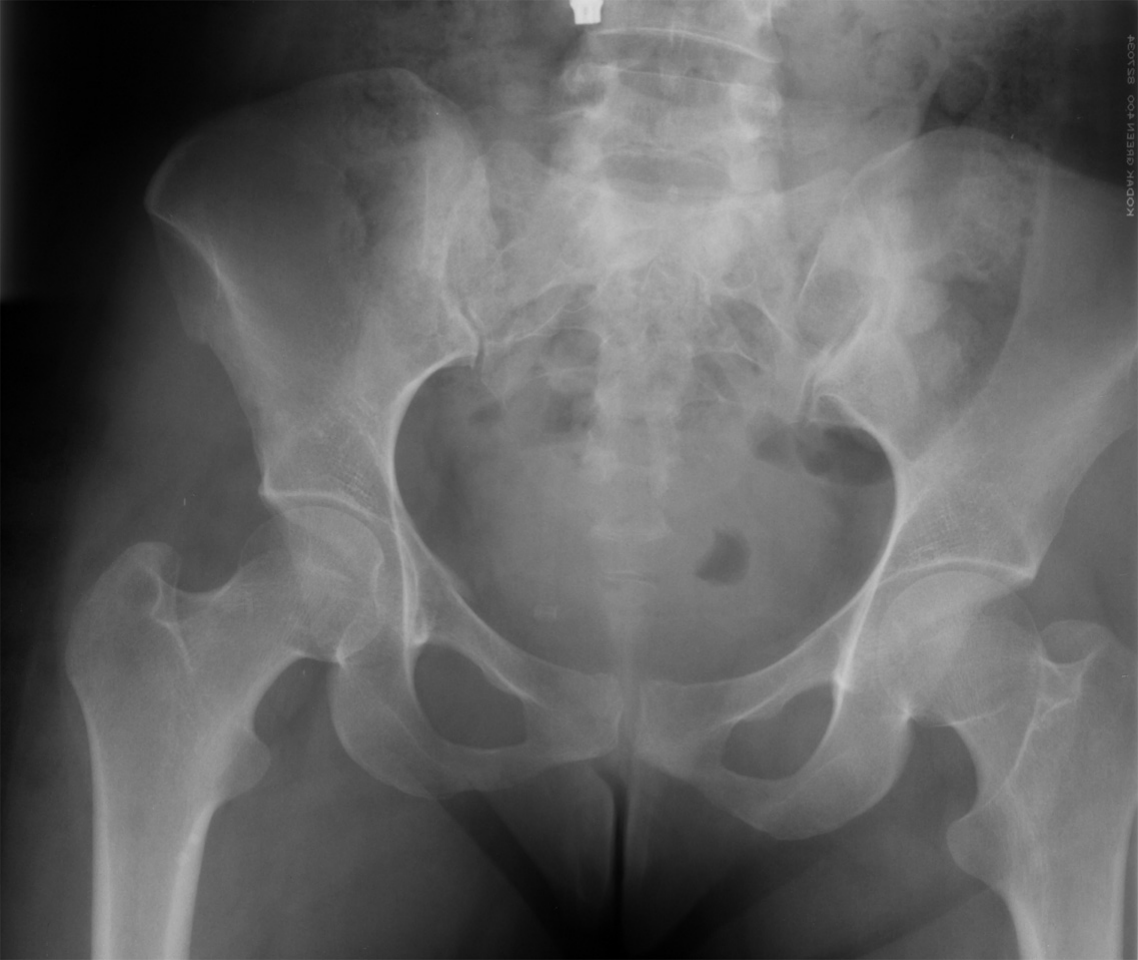
A vertical fracture line on the S1 vertebra can not be seen clearly on plain radiograph.

**Figure 2.**
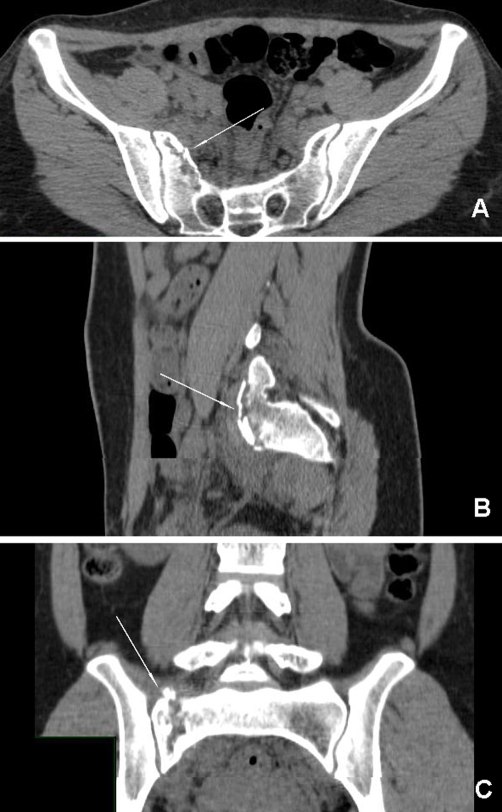
Axial **(A)**, sagittal **(B)** and coronal **(C)** reformatted CT images showing incomplete, fracture located on the superior and anterior part of the first sacral vertebra and no disruption of the right sacroiliac joint and the first foramina.

**Figure 3.**
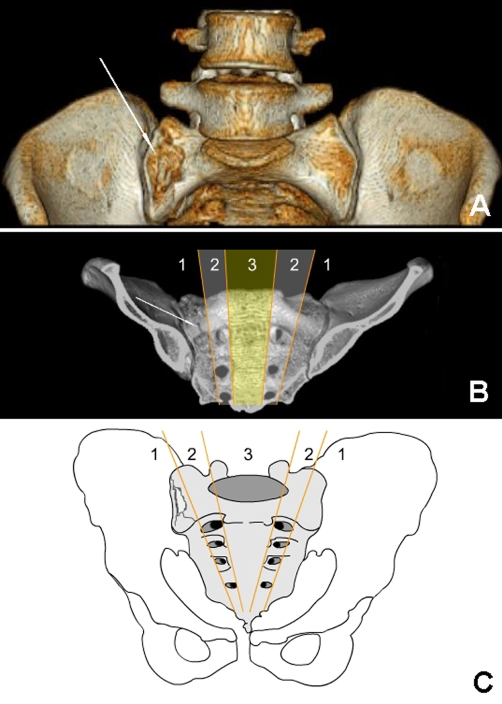
**(A)**: After 3 D reconstruction, it is obvious that the fracture is limited in zone I, **(B)**: The fracture is shown in the diagram of the 3 D imaging according to the Young & Burgess classification, **(C)**: Young & Burgess classification. (The figure was drawn by the author, K Soylu).

A 29-year-old Turkish female patient, who sat in front seat in the car, was unrestrained, and another car hit them from right front side of their vehicle. There was no loss of consciousness and hemodynamic remained stable on presentation to our emergency department. On admission, the patient complained of right-sided sacral and low back pain. Physical examination revealed considerable tenderness over the right superior gluteal region and excruciating pain during sacral and iliac compression. Sensorial and motor functions of both extremities were good during the neurological evaluation. There was also no urinary or anal incontinence. Plain radiographs of the cranium, chest, cervical and lumbosacral vertebral column and pelvis were taken to evaluate the potential injuries. There was no clear fracture line in her plain radiographs (Figure 1). Because of her considerable pain over the right sacral region, the patient was referred to multidetector computed tomography (CT) to better illustrate the pelvic bones. CT revealed incomplete, zone I fracture located on the superior and anterior part of the first sacral vertebra (Figure 2). On CT images, there were no abnormalities in the sacroiliac joint, symphisis pubis, superior-inferior pubic ramus, and first sacral foramina on the both side (Figure 3). Based on her fracture pattern the patient was allowed to ambulate with a crutch as tolerated and was discharged from the hospital for bed-rest following two days of observation.

## Discussion

Denis et al. classified the sacral fractures according to the anatomic location of the fracture, taking the potential neurological injury into consideration: zone I fractures in the region of the ala, zone II fractures in the region of the sacral foramina, and zone III fractures involving the central canal [2]. In view of this classification, our case was zone I fracture.

Vertical sacral fractures most commonly occur in alar or foraminal zones and they are essentially never isolated, always being associated with an anterior break in the pelvic ring [1,3]. In our case there was an alar sacral fracture without the other pelvic injuries.

The transverse fractures are seen frequently in patients with suicidal intent and have been referred to as the jumper's fracture [4]. Pelvic fracture classifications are usually based on the injury mechanism such as external rotation, lateral compression or vertical shear [1]. Uncommon sacral fractures usually occur by shear forces on the pelvic ring. Our patient was injured with the lateral compressive force. Such an unclassified isolated fracture of the sacrum can be explained by these force patterns, according to known mechanisms. Lateral compressive forces usually cause lateral compressive type (Young & Burgess) pelvic fractures such as type 1 lateral compression pelvic fractures [5]. According to our opinion, such an unclassified isolated fracture of the sacrum (S1) can be explained by severity of the lateral compressive forces. If there had been much more severity of this force in our case, it could have caused type 1 lateral compression pelvic fracture.

Type 1 lateral compression pelvic fractures are relatively common and they include impacted sacral and ipsilateral rami fractures [5]. Although our case was occurred with the same injury mechanism we did not see ipsilateral rami fractures with the impacted sacral fracture. Only a few cases, related with the isolated sacral fracture, have been reported in the literature [1,3,6-8]. To our knowledge, no isolated vertical zone I fracture of the first sacral vertebra which occurred with the lateral compression injury has been described previously.

Certain injuries such as bladder rupture, closed head injury, and traumatic hernia may be associated with lateral compression pelvic fractures [5,9]. In our case there was closed head injury which was not required any treatment.

Since the sacrum is usually obscured by pelvic bones and soft tissue shadows of the intra-abdominal organs, and fractures are rarely disintegrated, sacral fractures may be overlooked during radiological evaluation. So, we would like to emphasize that any suspect for sacral fractures in adults with a lateral compression injury mechanism should be confirmed by computed tomography.

These kinds of sacral fractures in adults may be overlooked easily. The key for diagnosis of the sacral fractures is being skeptic. Fracture of the sacrum should be suspected in the presence of sacral pain and tenderness.
